# (3*R*,3a*S*,6a*R*)-2,5-Dimethyl-3-(5-phenyl-2-thien­yl)perhydro­pyrrolo[3,4-*d*][1,2]oxazole-4,6-dione

**DOI:** 10.1107/S1600536808020102

**Published:** 2008-07-05

**Authors:** Mustafa Odabaşoğlu, Hamdi Özkan, Yılmaz Yıldırır, Orhan Büyükgüngör

**Affiliations:** a Department of Chemistry, Faculty of Arts and Sciences, Mehmet Akif Ersoy University, TR-15030 Burdur, Turkey; b Department of Chemistry, Faculty of Arts and Sciences, Gazi University, Ankara, Turkey; c Department of Physics, Faculty of Arts and Sciences, Ondokuz Mayıs University, TR-55139 Kurupelit Samsun, Turkey

## Abstract

The crystal structure of the title compound, C_17_H_16_N_2_O_3_S, exhibits intra­molecular C—H⋯S and inter­molecular C—H⋯S and C—H⋯O hydrogen bonds, C—S⋯N [S⋯N = 3.033 (2) Å and C—S⋯N = 142.76 (9)°] inter­actions, and C—H⋯π inter­actions; these inter­actions generate *S*(4), *S(*6) and *R*_2_^2^(14) ring motifs. The isoxazole ring adopts an envelope conformation, with the N atom displaced by 0.672 (2) Å from the plane of the other ring atoms. The thio­phene ring is oriented with respect to the succinimide and phenyl rings at dihedral angles of 40.03 (12) and 5.21 (13)°, respectively. The dihedral angle between the succinimide and phenyl rings is 39.38 (12)°.

## Related literature

For general background, see: Huisgen (1960 ▶); Black *et al.* (1975 ▶); Richman (2001 ▶); De Clercq (2002 ▶); Donadas *et al.* (2004 ▶); Merino *et al.* (2003 ▶); Chiacchio *et al.* (2003 ▶); Iannazzo *et al.* (2002 ▶). For related literature, see: Heaney *et al.* (2001 ▶). For ring motif details, see: Bernstein *et al.* (1995 ▶); Etter (1990 ▶).
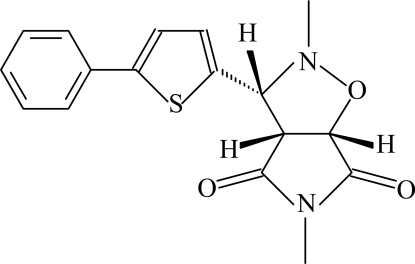


## Experimental

### 

#### Crystal data

C_17_H_16_N_2_O_3_S
*M* *_r_* = 328.38
Orthorhombic, 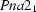

*a* = 12.7768 (7) Å
*b* = 10.9803 (6) Å
*c* = 11.1069 (9) Å
*V* = 1558.22 (17) Å^3^
*Z* = 4
Mo *K*α radiation
μ = 0.22 mm^−1^
*T* = 296 K
0.65 × 0.46 × 0.27 mm

#### Data collection

Stoe IPDSII diffractometer
Absorption correction: integration (*X-RED32*; Stoe & Cie, 2002 ▶) *T*_min_ = 0.889, *T*_max_ = 0.935
6395 measured reflections
3332 independent reflections
2899 reflections with *I* > 2σ(*I*)
*R*_int_ = 0.051

#### Refinement

*R*[*F* ^2^ > 2σ(*F* ^2^)] = 0.037
*wR*(*F* ^2^) = 0.099
*S* = 1.04
3332 reflections
221 parameters
1 restraint
H atoms treated by a mixture of independent and constrained refinement
Δρ_max_ = 0.22 e Å^−3^
Δρ_min_ = −0.22 e Å^−3^
Absolute structure: Flack (1983 ▶), 1451 Friedel pairs
Flack parameter = −0.13 (8)

### 

Data collection: *X-AREA* (Stoe & Cie, 2002 ▶); cell refinement: *X-AREA*; data reduction: *X-RED32* (Stoe & Cie, 2002 ▶); program(s) used to solve structure: *SHELXS97* (Sheldrick, 2008 ▶); program(s) used to refine structure: *SHELXL97* (Sheldrick, 2008 ▶); molecular graphics: *ORTEP-3 for Windows* (Farrugia, 1997 ▶); software used to prepare material for publication: *WinGX* (Farrugia, 1999 ▶).

## Supplementary Material

Crystal structure: contains datablocks I, global. DOI: 10.1107/S1600536808020102/hk2484sup1.cif

Structure factors: contains datablocks I. DOI: 10.1107/S1600536808020102/hk2484Isup2.hkl

Additional supplementary materials:  crystallographic information; 3D view; checkCIF report

## Figures and Tables

**Table 1:** Hydrogen-bond geometry (Å, °) *Cg*1 is the centroid of the S1/C7–C10 ring.

*D*—H⋯*A*	*D*—H	H⋯*A*	*D*⋯*A*	*D*—H⋯*A*
C6—H6⋯S1	0.93	2.68	3.097 (3)	108
C8—H8⋯S1^i^	0.93	3.00	3.887 (2)	160
C14—H14*B*⋯O2^i^	0.96	2.65	3.417 (4)	137
C13—H13⋯*Cg*1^ii^	1.01 (3)	2.96 (3)	3.875 (2)	152 (2)

Symmetry codes: (i) 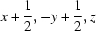; (ii) 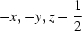.

**Fig. 1.**
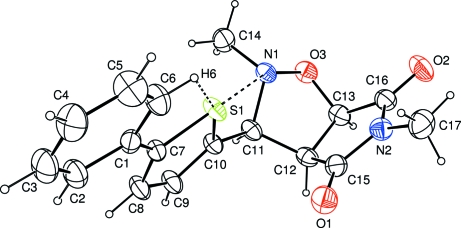
The molecular structure of the title molecule, with the atom-numbering scheme. Displacement ellipsoids are drawn at the 20% probability level. Hydrogen bonds are shown as dashed lines.

**Fig. 2.**
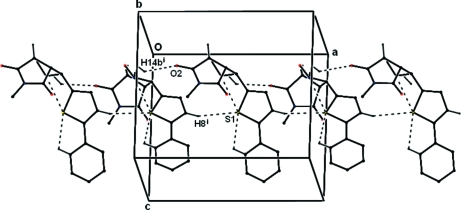
A partial packing diagram of (I), showing the formation of S(4), S(6) and R22(14) ring motifs along [100] [symmetry code: (i) x - 1/2, y, z]. Hydrogen bonds are shown as dashed lines. H atoms not involved in hydrogen bonding have been omitted for clarity.

**Fig. 3.**
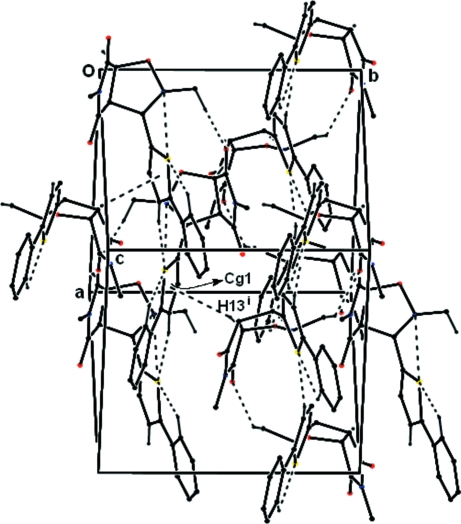
A packing diagram of (I) [symmetry code: (i) 1 - x, 1 - y, -z], where Cg1 is the centroid of ring (S1/C7-C10). Hydrogen bonds are shown as dashed lines. H atoms not involved in hydrogen bonding have been omitted for clarity.

## supplementary crystallographic information

### Comment

A general principle for the synthesis of five-membered rings was introduced in 1960 as 1,3-dipolar cycloaddition and turned out to be remarkably widespread (Huisgen, 1960, 1961). Because of easy 1,3-dipolar cycloaddition reactions to alkenes, alkynes, isocyanates, isothiocyanates, phospharanes, sulphenes and sulphynl compounds; nitrones are the important intermediates in synthetic organic chemistry (Black *et al.*,  1975). There has been an ever-increasing quest for modified nucleosides, due to their potential applications in antiviral and anticancer therapies (Richman, 2001; De Clercq, 2002; Donadas *et al.*,  2004). In a recent approach to modified nucleosides, the furanose ring has been replaced by other heterocyclic analogs (Merino *et al.*,  2003). Among these N and O containing heterocycles have emerged as important candidates, and have been shown to display useful anticancer and antiviral properties (Chiacchio *et al.*,  2003; Iannazzo *et al.*,  2002). The present work is part of a structural study of compounds of substited 2,5-dimethyl-4-(thiophen-2-yl)-tetrahydropyrrolo[3,4 -*c*]pyrrole-1,\3(2*H*,3aH)-dione systems with hydrogen-bond donors, and we report herein the crystal structure of the title compound, (I).

The overall view and atom-labelling of the molecule of (I) are displayed in Fig. 1. The thiophene ring is oriented with respect to succinimide and phenyl rings at dihedral angles of 40.03 (12)° and 5.21 (13)°, respectively. The dihedral angle between succinimide and phenyl rings is 39.38 (12)°. The isoxazole ring has envelope conformation, with N1 atom displaced by -0.672 (2) Å from the plane of the other ring atoms.

The hydrogen-bonding parameters are given in Table 1 and the packing arrangements of the molecules are illustrated in Figs. 2 and 3. Compound is stabilized by intramolecular C—H···S hydrogen bond and S···N heteroatom interactions [in C1—S1···N; S···N = 3.033 (2) Å, C1—S1···N = 142.76 (9) °], which form S(4) and S(6) motifs, and intermolecular C—H···S and C—H···O hydrogen bonds and C—H···π interactions. As shown in Fig. 2 the structure of the compound is made up of C8—H8···S1 and C14—H14B···O2 H-bonded polymeric bands of [C_12_H_14_N_2_O_3_S] molecules, which are nearly elongated along [100]. These polymeric chains are linked to each other and generate R_2_^2^(14) ring motifs (Bernstein *et al.*,  1995; Etter, 1990). The crystal packing is also stabilized by C13—H13···*Cg*1 interactions (Fig. 3, Table 1).

### Experimental

*N*-Methyl-*C*-(-5-Phenylthiophen) nitrone, (II), was prepared from 5-Phenylthiophenecarbaldehyde, *N*-methyl-hydroxylamine hydrochloride and sodium carbonate in CH_2_Cl_2_ according to the literature method (Heaney *et al.*,  2001). For the preparation of the title compound, (II) (657 mg, 3 mmol) and *N*-methylmaleimide (370 mg, 3.3 mmol) were dissolved in benzene (50 ml). The reaction mixture was refluxed for 12 h, and monitored by TLC. After evaporation of the solvent, the reaction mixture was separated by column chromatography, using the mixture of petroleum ether/ethyl acetate (2:1) as the eluant. The *cis*-isomer, (I), was recrystallized from CHCl_3_/n-hexane (mp: 452,9-454,4 K).

### Refinement

1451 Friedel pairs were averaged before the final refinement, but the absolute configuration could not be determined unambiguously, although Si atom is present. The methine H atoms, H11, H12 and H13, were located in difference syntheses and refined isotropically [C-H = 0.95 (3)-1.04 (3) Å; U_iso_(H) = 0.035 (6)-0.071 (10) Å^2^]. The remaining H atoms were positioned geometrically, with C-H = 0.93 and 0.96 Å for aromatic and methyl H, respectively, and constrained to ride on their parent atoms with U_iso_(H) = xU_eq_(C), where x = 1.5 for C14 methyl H and x = 1.2 for all other H atoms.

### Crystal data

**Table:**

C_17_H_16_N_2_O_3_S	*F*_000_ = 688
*M**_r_* = 328.38	*D*_x_ = 1.400 Mg m^−^^3^
Orthorhombic, *P**n**a*2_1_	Mo *K*α radiation λ = 0.71073 Å
Hall symbol: P 2c -2n	Cell parameters from 6395 reflections
*a* = 12.7768 (7) Å	θ = 1.8–28.0º
*b* = 10.9803 (6) Å	µ = 0.22 mm^−^^1^
*c* = 11.1069 (9) Å	*T* = 296 K
*V* = 1558.22 (17) Å^3^	Prism, colorless
*Z* = 4	0.65 × 0.46 × 0.27 mm

### Data collection

**Table:**

Stoe IPDSII diffractometer	3332 independent reflections
Monochromator: plane graphite	2899 reflections with *I* > 2σ(*I*)
Detector resolution: 6.67 pixels mm^-1^	*R*_int_ = 0.051
*T* = 296 K	θ_max_ = 27.5º
ω scan rotation method	θ_min_ = 2.5º
Absorption correction: integration(X-RED32; Stoe & Cie, 2002)	*h* = −13→16
*T*_min_ = 0.889, *T*_max_ = 0.935	*k* = −13→14
6395 measured reflections	*l* = −12→14

### Refinement

**Table:**

Refinement on *F*^2^	Hydrogen site location: inferred from neighbouring sites
Least-squares matrix: full	H atoms treated by a mixture of independent and constrained refinement
*R*[*F*^2^ > 2σ(*F*^2^)] = 0.037	*w* = 1/[σ^2^(*F*_o_^2^) + (0.0659*P*)^2^] where *P* = (*F*_o_^2^ + 2*F*_c_^2^)/3
*wR*(*F*^2^) = 0.099	(Δ/σ)_max_ < 0.001
*S* = 1.04	Δρ_max_ = 0.22 e Å^−^^3^
3332 reflections	Δρ_min_ = −0.22 e Å^−^^3^
221 parameters	Extinction correction: SHELXL97 (Sheldrick, 2008), Fc^*^=kFc[1+0.001xFc^2^λ^3^/sin(2θ)]^-1/4^
1 restraint	Extinction coefficient: 0.0122 (17)
Primary atom site location: structure-invariant direct methods	Absolute structure: Flack (1983)
Secondary atom site location: difference Fourier map	Flack parameter: −0.13 (8)

### Special details

**Table:**

Geometry. All esds (except the esd in the dihedral angle between two l.s. planes) are estimated using the full covariance matrix. The cell esds are taken into account individually in the estimation of esds in distances, angles and torsion angles; correlations between esds in cell parameters are only used when they are defined by crystal symmetry. An approximate (isotropic) treatment of cell esds is used for estimating esds involving l.s. planes.
Refinement. Refinement of F^2^ against ALL reflections. The weighted R-factor wR and goodness of fit S are based on F^2^, conventional R-factors R are based on F, with F set to zero for negative F^2^. The threshold expression of F^2^ > 2sigma(F^2^) is used only for calculating R-factors(gt) etc. and is not relevant to the choice of reflections for refinement. R-factors based on F^2^ are statistically about twice as large as those based on F, and R- factors based on ALL data will be even larger.

### Fractional atomic coordinates and isotropic or equivalent isotropic displacement parameters (Å^2^)

**Table:**

	*x*	*y*	*z*	*U*_iso_*/*U*_eq_	
C1	0.64659 (16)	0.19809 (17)	0.66560 (19)	0.0427 (4)	
C2	0.7443 (2)	0.1782 (2)	0.7188 (2)	0.0585 (6)	
H2	0.8048	0.1938	0.6749	0.070*	
C3	0.7523 (2)	0.1360 (3)	0.8346 (3)	0.0701 (7)	
H3	0.8181	0.1227	0.8680	0.084*	
C4	0.6641 (3)	0.1132 (3)	0.9022 (3)	0.0721 (8)	
H4	0.6696	0.0857	0.9811	0.087*	
C5	0.5678 (2)	0.1320 (3)	0.8504 (2)	0.0731 (8)	
H5	0.5075	0.1163	0.8948	0.088*	
C6	0.55911 (19)	0.1738 (2)	0.7341 (2)	0.0593 (6)	
H6	0.4930	0.1857	0.7011	0.071*	
C7	0.63920 (16)	0.24233 (18)	0.5415 (2)	0.0411 (4)	
C8	0.71619 (15)	0.2782 (2)	0.4653 (3)	0.0530 (5)	
H8	0.7866	0.2789	0.4864	0.064*	
C9	0.67952 (15)	0.3144 (2)	0.3508 (2)	0.0511 (5)	
H9	0.7236	0.3398	0.2891	0.061*	
C10	0.57421 (14)	0.30882 (18)	0.33939 (19)	0.0409 (4)	
C11	0.50963 (15)	0.3425 (2)	0.23306 (19)	0.0432 (4)	
C12	0.46103 (16)	0.4708 (2)	0.22929 (19)	0.0458 (4)	
C13	0.36143 (17)	0.4492 (2)	0.15649 (19)	0.0473 (5)	
C14	0.4393 (2)	0.1435 (2)	0.1799 (2)	0.0589 (6)	
H14A	0.3761	0.0963	0.1764	0.088*	
H14B	0.4884	0.1044	0.2327	0.088*	
H14C	0.4690	0.1498	0.1007	0.088*	
C15	0.42400 (17)	0.51840 (19)	0.3496 (2)	0.0490 (5)	
C16	0.27329 (17)	0.4855 (2)	0.2411 (2)	0.0506 (5)	
C17	0.2543 (2)	0.5584 (3)	0.4516 (3)	0.0716 (8)	
H17A	0.2708	0.6414	0.4711	0.086*	
H17B	0.2700	0.5072	0.5192	0.086*	
H17C	0.1812	0.5520	0.4326	0.086*	
N1	0.41573 (13)	0.26461 (15)	0.22512 (15)	0.0419 (4)	
N2	0.31576 (15)	0.52026 (17)	0.34890 (18)	0.0504 (4)	
O1	0.47725 (15)	0.55117 (17)	0.43306 (17)	0.0690 (5)	
O2	0.18093 (14)	0.48473 (19)	0.2184 (2)	0.0786 (6)	
O3	0.35719 (12)	0.32333 (14)	0.12785 (14)	0.0506 (4)	
S1	0.51891 (3)	0.25445 (5)	0.47066 (6)	0.04736 (14)	
H11	0.5520 (18)	0.3385 (19)	0.161 (2)	0.038 (5)*	
H12	0.511 (2)	0.535 (3)	0.191 (3)	0.068 (8)*	
H13	0.356 (2)	0.494 (3)	0.079 (3)	0.064 (8)*	

### Atomic displacement parameters (Å^2^)

**Table:**

	*U*^11^	*U*^22^	*U*^33^	*U*^12^	*U*^13^	*U*^23^
C1	0.0437 (10)	0.0377 (9)	0.0468 (11)	0.0072 (8)	−0.0082 (8)	−0.0047 (8)
C2	0.0489 (11)	0.0677 (15)	0.0590 (14)	0.0064 (10)	−0.0144 (11)	0.0013 (11)
C3	0.0682 (16)	0.0798 (17)	0.0622 (16)	0.0142 (13)	−0.0284 (14)	−0.0017 (13)
C4	0.085 (2)	0.0832 (17)	0.0478 (14)	0.0131 (15)	−0.0162 (13)	0.0040 (12)
C5	0.0745 (18)	0.096 (2)	0.0489 (14)	0.0006 (14)	0.0033 (14)	0.0095 (13)
C6	0.0491 (13)	0.0780 (16)	0.0508 (13)	0.0091 (10)	−0.0068 (10)	0.0064 (11)
C7	0.0322 (9)	0.0409 (9)	0.0501 (12)	0.0039 (8)	−0.0056 (8)	−0.0030 (7)
C8	0.0282 (8)	0.0666 (13)	0.0643 (13)	0.0027 (8)	−0.0018 (11)	0.0054 (14)
C9	0.0299 (9)	0.0670 (13)	0.0564 (12)	0.0011 (9)	0.0055 (9)	0.0079 (10)
C10	0.0305 (8)	0.0497 (10)	0.0426 (10)	0.0005 (7)	0.0039 (8)	0.0012 (8)
C11	0.0339 (9)	0.0595 (11)	0.0361 (9)	−0.0023 (8)	0.0046 (8)	0.0052 (8)
C12	0.0392 (9)	0.0555 (11)	0.0428 (10)	−0.0083 (9)	−0.0024 (8)	0.0111 (9)
C13	0.0448 (10)	0.0572 (11)	0.0398 (11)	−0.0034 (9)	−0.0074 (8)	0.0106 (8)
C14	0.0554 (13)	0.0613 (13)	0.0600 (14)	0.0043 (10)	−0.0034 (11)	−0.0127 (10)
C15	0.0496 (11)	0.0466 (10)	0.0507 (11)	0.0009 (9)	−0.0084 (10)	0.0050 (8)
C16	0.0437 (12)	0.0522 (11)	0.0559 (14)	−0.0022 (8)	−0.0037 (9)	0.0045 (9)
C17	0.0770 (17)	0.0676 (15)	0.0704 (19)	0.0072 (12)	0.0215 (14)	−0.0051 (13)
N1	0.0374 (8)	0.0523 (9)	0.0361 (8)	−0.0025 (7)	−0.0026 (6)	−0.0002 (7)
N2	0.0497 (10)	0.0516 (9)	0.0499 (10)	−0.0011 (8)	0.0039 (9)	0.0046 (7)
O1	0.0711 (12)	0.0742 (11)	0.0618 (11)	0.0082 (9)	−0.0242 (9)	−0.0139 (9)
O2	0.0387 (9)	0.0955 (13)	0.1016 (16)	0.0011 (8)	−0.0111 (9)	−0.0080 (12)
O3	0.0506 (9)	0.0618 (9)	0.0394 (7)	−0.0024 (7)	−0.0121 (6)	0.0002 (6)
S1	0.0287 (2)	0.0711 (3)	0.0423 (2)	−0.0033 (2)	−0.0012 (2)	0.0108 (2)

### Geometric parameters (Å, °)

**Table:**

C1—C6	1.378 (3)	C11—C12	1.540 (3)
C1—C2	1.398 (3)	C11—H11	0.97 (2)
C1—C7	1.464 (3)	C12—C15	1.511 (3)
C2—C3	1.371 (4)	C12—C13	1.526 (3)
C2—H2	0.9300	C12—H12	1.04 (3)
C3—C4	1.377 (5)	C13—O3	1.419 (3)
C3—H3	0.9300	C13—C16	1.520 (3)
C4—C5	1.374 (4)	C13—H13	1.00 (3)
C4—H4	0.9300	C14—N1	1.453 (3)
C5—C6	1.375 (4)	C14—H14A	0.9600
C5—H5	0.9300	C14—H14B	0.9600
C6—H6	0.9300	C14—H14C	0.9600
C7—C8	1.357 (3)	C15—O1	1.205 (3)
C7—S1	1.732 (2)	C15—N2	1.383 (3)
C8—C9	1.412 (4)	C16—O2	1.207 (3)
C8—H8	0.9300	C16—N2	1.369 (3)
C9—C10	1.353 (3)	C17—N2	1.447 (3)
C9—H9	0.9300	C17—H17A	0.9600
C10—C11	1.487 (3)	C17—H17B	0.9600
C10—S1	1.727 (2)	C17—H17C	0.9600
C11—N1	1.476 (3)	N1—O3	1.464 (2)
			
C6—C1—C2	117.4 (2)	C15—C12—C11	114.73 (17)
C6—C1—C7	122.11 (19)	C13—C12—C11	102.00 (17)
C2—C1—C7	120.5 (2)	C15—C12—H12	108.7 (15)
C3—C2—C1	121.1 (3)	C13—C12—H12	113.7 (15)
C3—C2—H2	119.5	C11—C12—H12	112.3 (16)
C1—C2—H2	119.5	O3—C13—C16	111.44 (18)
C2—C3—C4	120.8 (2)	O3—C13—C12	107.59 (17)
C2—C3—H3	119.6	C16—C13—C12	104.45 (18)
C4—C3—H3	119.6	O3—C13—H13	106.8 (16)
C5—C4—C3	118.5 (3)	C16—C13—H13	110.9 (17)
C5—C4—H4	120.7	C12—C13—H13	115.7 (17)
C3—C4—H4	120.7	N1—C14—H14A	109.5
C4—C5—C6	121.0 (3)	N1—C14—H14B	109.5
C4—C5—H5	119.5	H14A—C14—H14B	109.5
C6—C5—H5	119.5	N1—C14—H14C	109.5
C5—C6—C1	121.2 (2)	H14A—C14—H14C	109.5
C5—C6—H6	119.4	H14B—C14—H14C	109.5
C1—C6—H6	119.4	O1—C15—N2	124.4 (2)
C8—C7—C1	129.6 (2)	O1—C15—C12	127.4 (2)
C8—C7—S1	109.71 (17)	N2—C15—C12	108.27 (18)
C1—C7—S1	120.70 (16)	O2—C16—N2	124.9 (2)
C7—C8—C9	113.79 (18)	O2—C16—C13	126.4 (2)
C7—C8—H8	123.1	N2—C16—C13	108.67 (18)
C9—C8—H8	123.1	N2—C17—H17A	109.5
C10—C9—C8	113.7 (2)	N2—C17—H17B	109.5
C10—C9—H9	123.2	H17A—C17—H17B	109.5
C8—C9—H9	123.2	N2—C17—H17C	109.5
C9—C10—C11	127.94 (19)	H17A—C17—H17C	109.5
C9—C10—S1	110.08 (16)	H17B—C17—H17C	109.5
C11—C10—S1	121.97 (13)	C14—N1—O3	104.71 (16)
N1—C11—C10	110.73 (16)	C14—N1—C11	112.49 (17)
N1—C11—C12	101.59 (15)	O3—N1—C11	101.78 (14)
C10—C11—C12	118.22 (18)	C16—N2—C15	113.39 (19)
N1—C11—H11	112.3 (13)	C16—N2—C17	123.7 (2)
C10—C11—H11	109.5 (14)	C15—N2—C17	122.9 (2)
C12—C11—H11	104.2 (13)	C13—O3—N1	104.13 (14)
C15—C12—C13	105.14 (18)	C10—S1—C7	92.71 (10)
			
C6—C1—C2—C3	0.0 (3)	C13—C12—C15—O1	−177.3 (2)
C7—C1—C2—C3	−179.6 (2)	C11—C12—C15—O1	71.5 (3)
C1—C2—C3—C4	−0.6 (4)	C13—C12—C15—N2	1.9 (2)
C2—C3—C4—C5	0.9 (4)	C11—C12—C15—N2	−109.3 (2)
C3—C4—C5—C6	−0.6 (5)	O3—C13—C16—O2	−65.7 (3)
C4—C5—C6—C1	0.0 (4)	C12—C13—C16—O2	178.4 (2)
C2—C1—C6—C5	0.3 (4)	O3—C13—C16—N2	114.50 (19)
C7—C1—C6—C5	179.9 (2)	C12—C13—C16—N2	−1.4 (2)
C6—C1—C7—C8	175.0 (2)	C10—C11—N1—C14	−76.4 (2)
C2—C1—C7—C8	−5.4 (3)	C12—C11—N1—C14	157.21 (18)
C6—C1—C7—S1	−5.3 (3)	C10—C11—N1—O3	172.10 (16)
C2—C1—C7—S1	174.28 (17)	C12—C11—N1—O3	45.68 (17)
C1—C7—C8—C9	179.4 (2)	O2—C16—N2—C15	−177.0 (2)
S1—C7—C8—C9	−0.4 (3)	C13—C16—N2—C15	2.8 (3)
C7—C8—C9—C10	1.2 (3)	O2—C16—N2—C17	1.5 (4)
C8—C9—C10—C11	178.6 (2)	C13—C16—N2—C17	−178.8 (2)
C8—C9—C10—S1	−1.4 (3)	O1—C15—N2—C16	176.2 (2)
C9—C10—C11—N1	147.6 (2)	C12—C15—N2—C16	−3.0 (3)
S1—C10—C11—N1	−32.4 (2)	O1—C15—N2—C17	−2.2 (4)
C9—C10—C11—C12	−95.9 (3)	C12—C15—N2—C17	178.5 (2)
S1—C10—C11—C12	84.2 (2)	C16—C13—O3—N1	−86.75 (19)
N1—C11—C12—C15	84.5 (2)	C12—C13—O3—N1	27.2 (2)
C10—C11—C12—C15	−36.9 (3)	C14—N1—O3—C13	−163.37 (17)
N1—C11—C12—C13	−28.59 (19)	C11—N1—O3—C13	−46.07 (19)
C10—C11—C12—C13	−149.94 (17)	C9—C10—S1—C7	1.06 (18)
C15—C12—C13—O3	−118.85 (18)	C11—C10—S1—C7	−178.96 (17)
C11—C12—C13—O3	1.2 (2)	C8—C7—S1—C10	−0.40 (17)
C15—C12—C13—C16	−0.3 (2)	C1—C7—S1—C10	179.86 (16)
C11—C12—C13—C16	119.74 (18)		

### Hydrogen-bond geometry (Å, °)

**Table:**

*D*—H···*A*	*D*—H	H···*A*	*D*···*A*	*D*—H···*A*
C6—H6···S1	0.93	2.69	3.100 (2)	108
C8—H8···S1^i^	0.93	3.00	3.887 (2)	160
C14—H14B···O2^i^	0.96	2.65	3.417 (4)	137
C13—H13···Cg1^ii^	1.00 (3)	2.97 (3)	3.876 (2)	151 (2)

Symmetry codes: (i) *x*+1/2, −*y*+1/2, *z*; (ii) −*x*, −*y*, *z*−1/2.
